# Optimization and Validation of an HPLC–PDA Method for the Determination of (6S)-5-Methyltetrahydrofolate in Diverse Dietary Supplement Formulations

**DOI:** 10.3390/foods15071171

**Published:** 2026-03-31

**Authors:** Young-Jae Heo, Geun-Hee Cho, Tae-Woong Song, Su-Jong Kim, Ji-Hyun Im, Xiaolu Fu, June-Seok Lim, Min-Hye Kim, Hee-Jae Suh, Ok-Hwan Lee, Sun-Il Choi

**Affiliations:** 1 Department of Food Biotechnology and Environmental Science, Kangwon National University, Chuncheon 24341, Republic of Korea; 1204dudwo@naver.com (Y.-J.H.); wkdend@naver.com (G.-H.C.); stw902@naver.com (T.-W.S.); ijh108020@gmail.com (J.-H.I.); fuxiaolu2019@gmail.com (X.F.); dlawnstjr725@naver.com (J.-S.L.); minhye8733@naver.com (M.-H.K.); 2 Department of Food Science and Biotechnology, Kangwon National University, Chuncheon 24341, Republic of Korea; cfdesx@naver.com; 3 Department of Food Science, Research Center for Food and Bio Convergence, Sun Moon University, Asan 31460, Republic of Korea; suhhj@sunmoon.ac.kr

**Keywords:** (6S)-5-methyltetrahydrofolate, HPLC–PDA, method optimization, sample preparation, food additives

## Abstract

Analytical methods for (6S)-5-methyltetrahydrofolate (5-MTHF) have mainly been reported for specific formulation types, such as tablets, chewable tablets, powders, and liquid formulations, despite its increasing use in dietary supplement and health functional food formulations with diverse matrix compositions. In this study, high-performance liquid chromatography with photodiode array detection analytical conditions and sample preparation approaches were systematically compared and optimized to improve applicability across different formulation matrices. Chromatographic performance was evaluated under standard solution conditions, followed by a comparative assessment of sample preparation conditions in tablet, chewable tablet, powder, and liquid formulations. An ultrasonic pretreatment approach without thermal treatment provided consistent recovery and repeatability across all the tested formulations. The optimized analytical conditions showed linear detector response (R^2^ = 0.9992), precision with relative standard deviation values of 0.52–4.06%, recoveries of 81.84–102.56%, and an estimated expanded measurement uncertainty of approximately 10%. These results indicate that the optimized HPLC–PDA analytical conditions are applicable to the determination of 5-MTHF across diverse dietary supplement and health functional food formulations.

## 1. Introduction

Folic acid and its bioactive forms are among the most widely used nutritional ingredients in fortified foods, incorporated into a wide range of dietary supplements and health functional food products, particularly those fortified with vitamins and minerals such as folic acid and 5-MTHF [1,2,3].

Folic acid is a representative water-soluble B-group vitamin that is essential for nucleic acid synthesis and cell division [2]. Owing to its critical role in fetal development during early pregnancy, folic acid is widely used as a key fortification ingredient in foods and health functional foods. Accordingly, its use and analytical control are regulated within national food regulatory frameworks [3].

In recent years, biologically active forms of folate with improved metabolic efficiency have attracted increasing attention [4]. Among these, (6S)-5-methyltetrahydrofolate (5-MTHF) can be directly utilized without requiring additional enzymatic reduction in vivo and has been reported as an alternative capable of mitigating interindividual variability in folate metabolism [5,6,7]. Consequently, 5-MTHF has been increasingly incorporated into dietary supplements and health functional food products, with formulation types expanding to include tablets, chewable tablets, powders, and liquid formulations [8]. In the present study, we focus on 5-MTHF contained in these dietary supplements and health functional food formulations rather than in general food matrices.

With the growing application of 5-MTHF, there is an increasing demand for analytical methods capable of reliably determining this compound in complex supplement matrices, taking into account its higher susceptibility to thermal, oxidative, and photolytic degradation compared with folic acid [9,10,11,12]. However, most existing studies have focused on analytical methods tailored to specific formulations or single matrices, while reports describing approaches broadly applicable across diverse formulation types remain limited [13,14]. In addition, some reported methods involve labor-intensive pretreatment procedures or include heating and reduction steps, which may limit their practical applicability in routine laboratory analysis. Accordingly, this study systematically evaluated previously reported HPLC–PDA analytical conditions and sample pretreatment procedures for the determination of 5-MTHF in various dietary supplements and health functional food formulations. Rather than proposing a new analytical principle, we focused on optimizing and harmonizing representative HPLC–PDA methods, including an official MFDS procedure and two widely cited literature approaches, so that a single analytical workflow can be applied across multiple dosage forms (tablets, chewable tablets, powders, and liquid formulations). In addition, we extend previous work by providing a quantitative evaluation of measurement uncertainty for the optimized method, offering practical information on the reliability of 5-MTHF determinations for routine quality control and regulatory monitoring.

## 2. Materials and Methods

### 2.1. Chemicals and Reagents

(6S)-5-Methyltetrahydrofolate calcium salt (≥95.0%) was obtained from HanDom Chemical Co., Ltd. (Dalian, China), and (6S)-5-methyltetrahydrofolate glucosamine salt (Quatrefolic®, diastereoisomeric purity ≥ 99.0%) was purchased from Gnosis by Lesaffre (Pisticci, Italy). Acetonitrile (≥99.0%) and methanol (≥99.0%) were obtained from JT Baker (Phillipsburg, NJ, USA) and used for chromatographic analysis. All other reagents used for mobile phase preparation and sample pretreatment were of analytical grade.

### 2.2. Sample Matrices

Representative formulation types commonly used in dietary supplements and health functional food products were selected as sample matrices to evaluate formulation-dependent analytical behavior. The evaluated formulations comprised four dosage forms (tablets, chewable tablets, powders, and liquid), and one commercial product was selected for each dosage form (a total of four products) from the Korean market and used as a representative test sample for that matrix type. The objective of this evaluation was to compare formulation-dependent analytical behavior rather than to assess product variability within the same dosage form.

### 2.3. Optimization of HPLC Instrument Conditions

To optimize HPLC–PDA analytical conditions applicable across diverse formulation matrices, three previously reported analytical methods were selected as initial candidate conditions and systematically evaluated in a stepwise manner. Optimization was initiated under standard solution conditions to exclude matrix-related effects and to allow for direct comparison of intrinsic chromatographic performance. The candidate conditions consisted of an HPLC–PDA procedure described in the Food Additives Code of Korea and two literature-reported methods developed for the determination of 5-MTHF in dietary supplements and health functional food matrices [15], which differ in mobile phase composition, buffer usage, and overall procedural complexity. The chromatographic settings (column type, mobile phases, and gradient program) of these three candidate methods are summarized in Table 1 and described in detail in the corresponding references [15,16,17], whereas the final optimized HPLC–PDA conditions, including column type, mobile phases, gradient, flow rate, column temperature, run time, and detection wavelength, are provided in the latter part of this section. Calibration linearity, peak shape, retention time reproducibility, signal stability during repeated injections, and practical operability were employed as primary optimization criteria. Practical operability was considered by favoring buffer-free mobile phases that can be prepared from common solvents, moderate total run times compatible with routine batch analysis, and simple gradient programs that can be implemented on standard HPLC–PDA systems without special hardware or extensive equilibration steps. Based on this integrated evaluation, HPLC–PDA analytical conditions suitable for subsequent applicability assessment and method validation across various formulation matrices were established.

### 2.4. Optimization of Sample Preparation Procedures

Sample preparation procedures were established based on three previously reported extraction approaches, including an official procedure described in the Food Additives Code of Korea and two literature-based strategies [15]. All procedures were evaluated under identical optimized HPLC–PDA analytical conditions to allow direct comparison of extraction characteristics and analytical performance across different formulation matrices.

For the MFDS-based approach, a 1.5% (*w*/*v*) sodium sulfite solution was used as the extraction solvent. Samples were subjected to ultrasonic extraction for 20 min, followed by centrifugation at 2000× *g* for 10 min. The resulting supernatant was filtered through a 0.2 µm membrane filter prior to HPLC analysis. During ultrasonic extraction and any water bath heating steps evaluated, sample tubes were tightly capped and wrapped with aluminum foil, and the surface of the water bath was also covered with foil to minimize light exposure. After extraction and filtration, all sample solutions were kept in capped tubes and analyzed within a short timeframe under ambient laboratory lighting, avoiding direct exposure to strong light sources. In the procedure reported by Liu et al. [16], extraction was performed using a mixed solvent consisting of 1% acetic acid and 5% ascorbic acid (2:8, *v*/*v*). Samples were heated in a water bath at 95 °C for 30 min and subsequently filtered through a 0.45 µm membrane filter prior to analysis. For the ultrasonic extraction approach described by Alshishani et al. [17], a 1% ascorbic acid solution was used as the extraction solvent, with ultrasonic treatment applied for 30 min. After extraction, samples were centrifuged at 6000× *g* for 15 min, and the resulting supernatant was collected for analysis.

### 2.5. Method Validation

The optimized HPLC–PDA analytical conditions were validated for the determination of 5-MTHF in dietary supplements and health functional food matrices in accordance with the International Council for Harmonisation (ICH) Q2(R2) guideline and the recommendations of AOAC INTERNATIONAL [18,19]. The validation parameters evaluated included specificity, linearity, limit of detection (LOD), limit of quantification (LOQ), precision, accuracy, and matrix effects. Specificity was assessed by comparing chromatograms obtained from blank matrices, 5-MTHF standard solutions, and samples containing 5-MTHF for each formulation type. Linearity was evaluated using 5-MTHF standard solutions over a concentration range of 6.25–100 μg/mL, with calibration curves constructed by linear regression analysis and assessed based on the correlation coefficient (R^2^). The LOD and LOQ were calculated using a calibration curve-based approach according to the ICH guideline, based on the equations LOD = 3.3σ/S and LOQ = 10σ/S, where σ represents the standard deviation of the response, and S denotes the slope of the calibration curve. Precision was evaluated as repeatability under identical analytical conditions and assessed as intra-day and inter-day precision, with results expressed as relative standard deviation (RSD). Accuracy was determined by recovery experiments conducted at three concentration levels (12.5, 25, and 50 μg/mL) by spiking known amounts of 5-MTHF into each formulation matrix. Matrix effects were assessed by comparing the peak areas of 5-MTHF obtained from standard solutions with those from matrix-spiked samples at the same nominal concentration and expressed as relative response (%) relative to the corresponding standard solutions.

### 2.6. Measurement Uncertainty

Measurement uncertainty was evaluated to assess the reliability of the results obtained using the optimized HPLC–PDA analytical conditions. The uncertainty assessment was performed in accordance with the Eurachem/CITAC guidelines [20]. An uncertainty budget was established by considering the major sources of uncertainty arising throughout the analytical procedure. The uncertainty components included variability associated with sample preparation (*U*_prep_), uncertainty related to the purity of the reference material (*U*_RM_), uncertainty arising from standard solution preparation (*U*_std_), uncertainty associated with calibration curve construction (*U*_cal_), and variability due to repeatability (*U*_rep_). All uncertainty components were assumed to be independent. The combined standard uncertainty (*u*_c_) was calculated as the square root of the sum of the squares of the individual uncertainty components. The expanded uncertainty (*U*) was obtained by applying a coverage factor of *k* = 2, corresponding to an approximate confidence level of 95%. In addition, the relative contribution of each individual uncertainty component was calculated to evaluate its impact on the overall measurement uncertainty.

## 3. Results and Discussion

### 3.1. Optimization of HPLC Instrument Conditions

Under standard solution conditions, three previously reported HPLC–PDA analytical conditions for the determination of 5-MTHF were evaluated to optimize chromatographic performance, and their chromatographic characteristics are summarized in Table 1. All evaluated conditions exhibited satisfactory linearity, with correlation coefficients (R^2^) of 0.9984 for MFDS [15], 0.9997 for Liu et al. [16], and 0.9990 for Alshishani et al. [17], confirming their suitability for the quantitative determination of 5-MTHF under standard solution conditions. Among the evaluated conditions, those reported by Liu et al. [16] showed the highest linearity (R^2^ = 0.9997) together with consistent peak responses, indicating superior calibration reproducibility. In addition, the use of a simple mobile phase system composed of 0.1% trifluoroacetic acid in water and methanol, without buffer components, reduced procedural complexity and enhanced robustness across different HPLC–PDA systems.

Based on this comparative evaluation, the optimized chromatographic conditions were established using a reversed-phase C_18_ column (Capcell Pak C_18_ UG120, 4.6 mm × 250 mm, 5 µm; Shiseido, Tokyo, Japan). The column temperature was maintained at 32 °C with a flow rate of 1.0 mL/min, an injection volume of 40 µL, and PDA detection at 280 nm. The mobile phase consisted of water containing 0.1% (*v*/*v*) trifluoroacetic acid (A) and methanol (B). The gradient program was as follows: 0.0–1.0 min, 90% to 84.8% A; 1.0–18.0 min, 84.8% to 84.5% A; 18.0–18.01 min, 84.5% to 40% A; 18.01–20.0 min, 40% A; 20.0–20.01 min, 40% to 90% A; and 20.01–25.0 min, 90% A.

**Figure 1:**
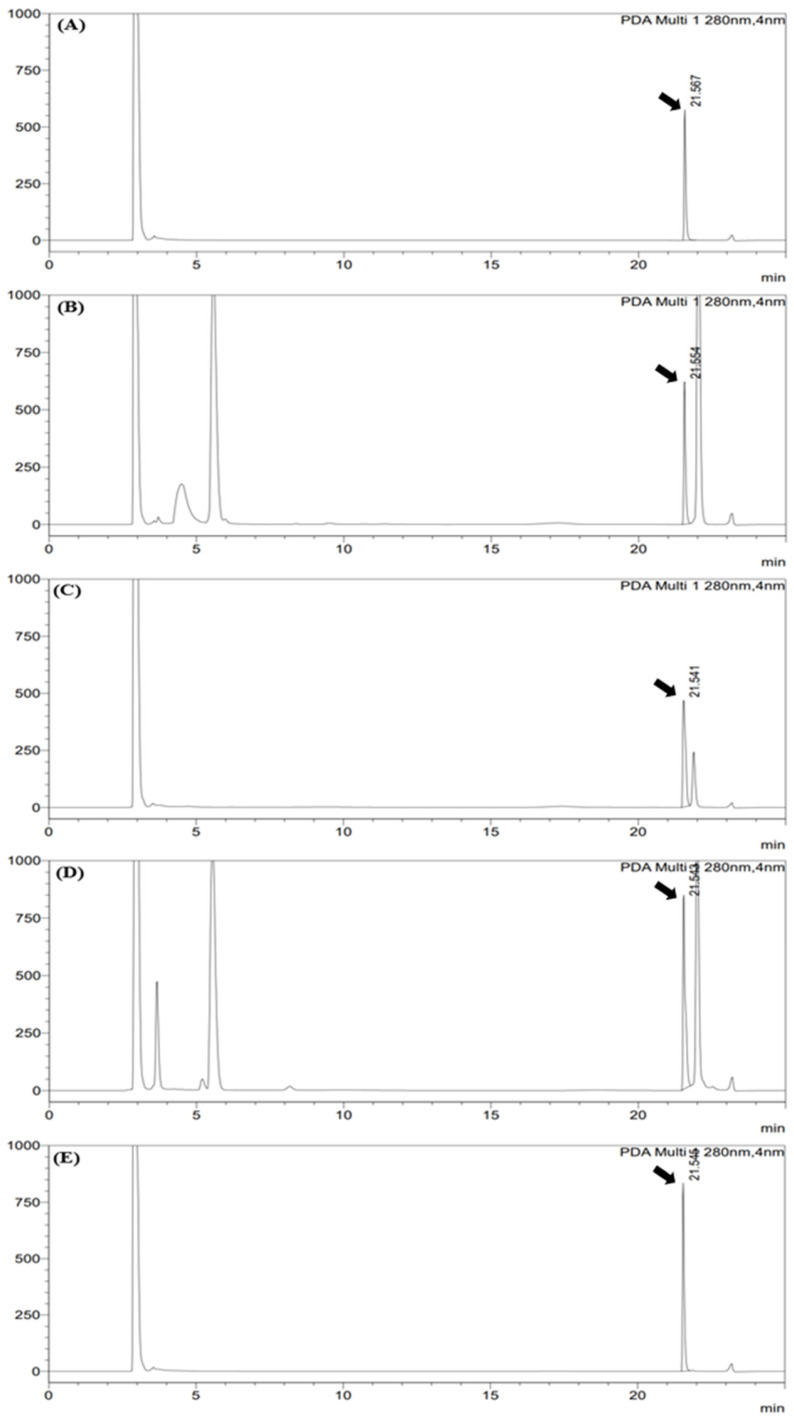
HPLC–PDA chromatograms of 5-MTHF at an equivalent concentration in different formulation matrices under the optimized chromatographic conditions (detection at 280 nm): (**A**) 5-MTHF standard solution, (**B**) tablet, (**C**) chewable tablet, (**D**) powder, and (**E**) liquid formulation. The 5-MTHF peak is indicated by arrows in each chromatogram.

The optimized HPLC–PDA analytical conditions were subsequently applied to tablet, chewable tablet, powder, and liquid formulations to evaluate applicability across different formulation matrices. In all formulations, 5-MTHF was consistently detected at a retention time of 21.55 ± 0.01 min (mean ± SD, *n* = 5; one standard solution and four formulation matrices), with retention time variation within 0.1 min among matrices, indicating satisfactory chromatographic reproducibility. Blank chromatograms of all formulation matrices showed no interfering peaks near the retention time of 5-MTHF, whereas samples containing 5-MTHF exhibited a clear and well-defined single peak regardless of formulation type (Figure 1). The resolution between the 5-MTHF peak and adjacent matrix-derived peaks exceeded 1.5 for all formulations, confirming baseline separation. Repeat injections for each formulation resulted in relative standard deviations of peak areas below 3%, demonstrating stable signal reproducibility despite differences in matrix composition. Differences in absolute peak areas were observed among formulations at the same nominal concentration, attributable to variations in excipient composition and matrix complexity, which can influence analyte response in chromatographic analysis [21]. Matrix-dependent signal variation was further evaluated quantitatively in the subsequent matrix effect assessment. Overall, the optimized HPLC–PDA analytical conditions provided stable chromatographic performance and sufficient peak resolution across formulation matrices with distinct physical characteristics, supporting their application as common analytical conditions for subsequent comparison of sample preparation procedures and method validation in accordance with ICH guidelines [18].

### 3.2. Optimization of Sample Preparation Procedures

To optimize sample preparation across formulation matrices, three pretreatment procedures differing in reducing agent composition and treatment conditions were evaluated, and the corresponding recovery and precision results are summarized in Table 2. The evaluated procedures were derived from the Food Additives Code of Korea [15], a literature-reported approach incorporating high-temperature heating [16], and an ultrasonic extraction-based approach [17].

The extraction conditions described by Liu et al. [16] yielded recovery values of 95.03–101.23% with relative standard deviations (RSDs) of 0.35–4.55% across all formulation matrices. However, this procedure involves a heating step at 95 °C, which may represent a potential limitation with respect to the chemical stability of 5-MTHF. Previous studies have demonstrated that 5-MTHF is highly sensitive to elevated temperatures, exhibiting a marked decrease in residual levels above 85 °C and a reduction to below half at 100 °C [9,10]. Moreover, compared with folic acid, 5-MTHF undergoes more rapid thermal degradation, with degradation kinetics accelerating substantially above 40 °C [11]. Although high recoveries were obtained under the high-temperature conditions, recovery alone does not directly reflect chemical stability. Thermal degradation and reductive regeneration may occur simultaneously, yielding an apparent net recovery. Therefore, high-temperature conditions require careful consideration when analytical robustness and long-term reproducibility are prioritized.

The ultrasonic extraction approach reported by Alshishani et al. [17] showed overall high extraction efficiency, with recovery values ranging from 94.49% to 107.80%. Nevertheless, a tendency toward recoveries exceeding 100% was observed under certain conditions. Ascorbic acid functions as an effective reducing agent but simultaneously creates an acidic environment in which redox-related transformations of 5-MTHF have been reported [22,23]. Recoveries exceeding 100% under acidic reducing conditions are therefore interpreted as apparent over-recovery, potentially arising from redox-related interconversion or enhanced matrix–analyte interactions, rather than reflecting true quantitative accuracy [22].

In contrast, the MFDS-based procedure employs sodium sulfite as a reducing agent under relatively mild conditions (approximately pH 5–6). These near-neutral conditions may mitigate matrix-dependent variability associated with acidic environments [12] and have been reported to delay the oxidative interconversion of reduced folates, thereby enhancing structural stability compared with acidic conditions [22]. In addition, this procedure is conducted under room-temperature ultrasonication, thereby avoiding thermal degradation pathways that are accelerated at temperatures above 40 °C [11]. Under low-temperature conditions, oxidative reactions are considered the primary degradation pathway of 5-MTHF, for which moderate reductive stabilization may be sufficient [10]. The lower recovery at the highest spiking level (50 μg/mL; 82.49%) under MFDS [15] conditions is likely due to reduced extraction efficiency from particle aggregation during ultrasonication, rather than chemical degradation [22]. This is consistent with the more stable recovery range observed at lower concentrations (84.28–97.66%). Overall, the MFDS-based ultrasonic approach was identified as the most suitable for subsequent validation. It operates under low-temperature, near-neutral conditions, thereby minimizing thermal degradation risks while providing acceptable recovery and repeatability across diverse formulation matrices.

### 3.3. Method Validation

The optimized HPLC–PDA analytical conditions were validated in accordance with the ICH Q2(R2) guideline with respect to specificity, linearity, limit of detection (LOD), limit of quantification (LOQ), precision, accuracy, and matrix effects [18]. Validation was performed across different formulation matrices, including tablet, chewable tablet, powder, and liquid formulations. All validation experiments were performed in triplicate, satisfying the minimum requirements specified in the ICH Q2(R2) guideline. Calibration curves were constructed using multiple concentration levels across the evaluated concentration range. Robustness testing was not separately evaluated, as the primary objective of this study was to optimize and harmonize chromatographic conditions and sample preparation procedures applicable across diverse formulation matrices rather than to assess deliberate variations in individual analytical parameters. Stability studies, such as bench-top or freeze–thaw stability, were also not included, since the analytical workflow was designed for immediate extraction and analysis under controlled laboratory conditions, consistent with routine quality control practices. Light exposure during sample preparation was minimized by wrapping tubes and water baths with aluminum foil and keeping samples capped until analysis, which helped reduce the risk of photodegradation of 5-MTHF under the laboratory conditions used in this study.

#### 3.3.1. Specificity

Specificity was evaluated by comparing chromatograms obtained from blank matrices, 5-MTHF standard solutions, and samples spiked with 5-MTHF for each formulation type. For all formulations, no interfering peaks were detected near the retention time of 5-MTHF (approximately 21.5 min). In addition, the resolution between the 5-MTHF peak and adjacent peaks exceeded 1.5 under all analytical conditions, indicating sufficient chromatographic separation and selective determination of 5-MTHF in complex formulation matrices.

#### 3.3.2. Linearity, LOD, and LOQ

Calibration curves constructed using 5-MTHF standard solutions over the concentration range of 6.25–100 μg/mL exhibited good linearity under both intra-day and inter-day conditions, with correlation coefficients (R^2^) of 0.9992. These results indicate a stable linear relationship between analyte concentration and detector response across the investigated range (Table 3). The LOD and LOQ were determined using a calibration curve-based approach. Under inter-day conditions, the LOD and LOQ were 0.15 μg/mL and 0.45 μg/mL, respectively, whereas under intra-day conditions, the corresponding values were 0.42 μg/mL and 1.26 μg/mL. These sensitivity levels are sufficient for the quantitative determination of 5-MTHF across the evaluated formulation matrices. The LOD and LOQ values were calculated from the calibration curve according to the ICH guideline using the equations LOD = 3.3σ/S and LOQ = 10σ/S, where σ represents the standard deviation of the response, and S denotes the slope of the calibration curve. The lower LOD and LOQ values observed under inter-day conditions are attributed to a lower σ estimated from the inter-day calibration data set, rather than an actual increase in instrumental sensitivity.

#### 3.3.3. Precision

Precision was assessed as intra-day and inter-day repeatability through repeated analyses. Under intra-day conditions, relative standard deviations were in the ranges of 1.04–1.84% for tablets, 0.54–1.09% for chewable tablets, 0.52–0.99% for powders, and 0.63–1.61% for liquid formulations. Inter-day precision results showed relative standard deviations of 2.40–4.06% for tablets, 1.93–3.93% for chewable tablets, 0.84–0.90% for powders, and 3.00–3.88% for liquid formulations. These results indicate consistent repeatability of the optimized analytical conditions across all formulation matrices.

#### 3.3.4. Accuracy

Accuracy was evaluated by recovery experiments performed at three spiking levels (12.5, 25, and 50 μg/mL) for each formulation matrix. Under inter-day conditions, recoveries were in the ranges of 84.87–96.44% for tablets, 90.27–95.64% for chewable tablets, 88.33–98.39% for powders, and 100.04–101.39% for liquid formulations. Under intra-day conditions, recoveries were in the ranges of 89.40–100.41% for tablets, 91.35–97.74% for chewable tablets, 81.84–95.52% for powders, and 100.90–102.56% for liquid formulations. These results demonstrate acceptable accuracy across formulation matrices despite differences in matrix composition. Overall, the recoveries obtained across all formulation matrices (approximately 82–103%) fell within the 80–110% target recovery range specified in AOAC Standard Method Performance Requirements for this concentration level.

#### 3.3.5. Matrix Effect

Matrix effects were evaluated by comparing the analytical responses of 5-MTHF in standard solutions with those obtained from matrix-spiked samples at the same nominal concentration. Relative responses across formulation matrices ranged from approximately 81.84% to 102.56%. According to commonly accepted criteria for matrix effect evaluation in chromatographic analysis, relative responses within ±20% of the corresponding standard solution are generally considered acceptable for quantitative determination [21]. A comparatively lower response was observed for powder formulations, whereas liquid formulations exhibited responses similar to those of standard solutions [21]. The lower response observed for powder formulations is interpreted as being associated with physical matrix characteristics, such as particle aggregation during extraction, rather than chemical degradation of 5-MTHF. Despite these variations, repeatability remained stable across all matrices, and the observed matrix effects were not considered to compromise the applicability of the optimized HPLC–PDA analytical conditions for quantitative analysis.

### 3.4. Measurement Uncertainty

**Figure 2:**
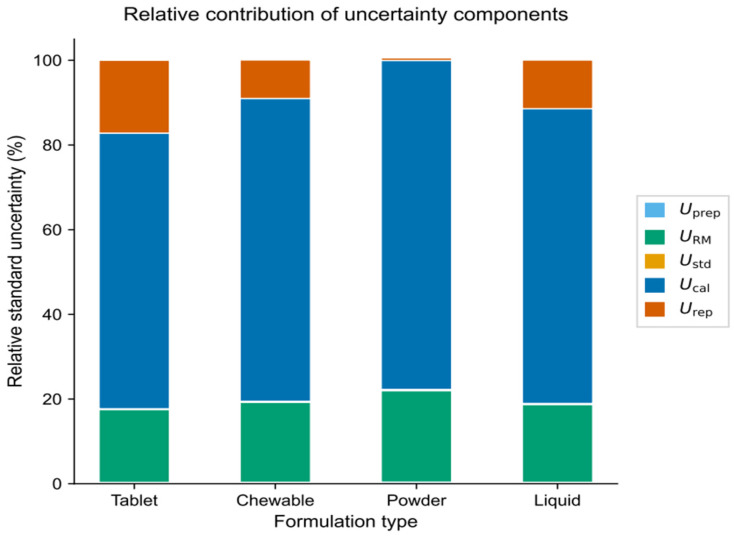
Contribution of each individual source of uncertainty in 5-MTHF determination. *U*_prep_, sample preparation; *U*_RM_, reference material; *U*_std_, standard stock solution; *U*_cal_, calibration curve; and *U*_rep_, repeatability. Some components show limited visibility due to their relatively small contribution.

Measurement uncertainty characterizes the dispersion of values that can reasonably be attributed to the measurand and represents a key parameter for evaluating the reliability of analytical results [24]. In this study, measurement uncertainty was estimated to assess the reliability of quantitative results obtained for 5-MTHF using the optimized HPLC–PDA analytical conditions, and the relative contribution of individual uncertainty components was further examined (Table 4, Figure 2). An uncertainty budget was established in accordance with the Eurachem/CITAC guideline by considering five major sources of uncertainty: sample preparation (*U*_prep_), reference material purity (*U*_RM_), standard solution preparation (*U*_std_), calibration curve construction (*U*_cal_), and repeatability (*U*_rep_) [19]. All uncertainty components and the expanded uncertainty (*U*) are expressed as relative standard uncertainties (dimensionless fractions), so that *U* = 0.0985 corresponds to an expanded uncertainty of approximately 9.85%. A bottom-up approach was applied, in which each uncertainty component was individually estimated and subsequently combined [24]. Contribution analysis revealed that *U*_cal_ (0.0397) was the dominant contributor to the overall measurement uncertainty, indicating that variability in detector response at low concentration levels and residuals associated with the regression model exerted the greatest influence on quantitative results. In contrast, the contributions from *U*_prep_ (0.00271) and *U*_rep_ (0.0206) were comparatively lower, reflecting consistent sample handling and analytical repeatability across formulation matrices. Similar uncertainty profiles have been reported in recent HPLC-DAD-based food analysis studies, in which calibration-related uncertainty was also identified as a major contributor [25,26]. The combined standard uncertainty (*u*_c_) was calculated using the root sum of squares of the individual uncertainty components, and the expanded uncertainty (*U*) was obtained by applying a coverage factor of *k* = 2, corresponding to an approximate confidence level of 95%. The final expanded uncertainty of the optimized analytical conditions was determined to be U = 0.0985. Expanded uncertainties of the order of approximately 10% are generally considered acceptable for quantitative analysis of dietary supplements and health functional food matrices, particularly for HPLC-based methods applied to complex formulation systems [26,27]. Recent studies have also reported expanded uncertainties below approximately 16–20% for similar dietary supplement and functional food analyses [26,27], supporting the validity of the uncertainty level obtained in this study. The dominance of calibration-related uncertainty further suggests that potential reduction in overall measurement uncertainty would primarily depend on improving calibration robustness, such as increasing the number of calibration points or reducing variability at lower concentration levels, rather than modifications to sample preparation or instrumental repeatability. Overall, the optimized HPLC–PDA analytical conditions provide reliable quantitative determination of 5-MTHF across diverse formulation matrices, with a level of measurement uncertainty suitable for regulatory monitoring and routine quality control applications. Each uncertainty component was estimated based on in-house validation data following the Eurachem guidance on quantifying uncertainty in analytical measurement. The sample preparation component (*U*_prep_) was derived from replicate extraction experiments, the reference material component (*U*_RM_) from the certified purity and its associated uncertainty, the standard solution component (*U*_std_) from weighing and volumetric operations, the calibration component (*U*_cal_) from the regression parameters of the calibration curve, and the repeatability component (*U*_rep_) from within-day precision studies. The combined standard uncertainty (*u*_c_) was calculated using the root-sum-of-squares method:

$$u_{c}=\surd ({u_{prep}}^{2}+{u_{RM}}^{2}+{u_{std}}^{2}+{u_{cal}}^{2}+{u_{rep}}^{2})$$

The expanded uncertainty (*U*) was obtained by multiplying the combined standard uncertainty by a coverage factor (*k* = 2):

$$U=k\times u_{c}$$

## 4. Conclusions

This study optimized an HPLC–PDA analytical strategy for the determination of 6S-5-methyltetrahydrofolate (5-MTHF) in dietary supplements, including health functional foods, encompassing diverse formulation matrices (tablets, chewable tablets, powders, and liquids) using a single harmonized workflow without matrix-specific adjustments. Through systematic comparison of previously reported chromatographic conditions and sample preparation approaches, analytical conditions applicable across tablet, chewable tablet, powder, and liquid formulations were established. Among the evaluated sample preparation approaches, the ultrasonic extraction conditions described in the Food Additives Code of Korea were found to provide consistent recovery and repeatability across all formulation matrices. These conditions avoid a heating step and are therefore compatible with the thermal sensitivity of 5-MTHF while maintaining procedural simplicity and applicability to formulations with different physical characteristics. The optimized HPLC–PDA analytical conditions were validated in accordance with the ICH Q2(R2) guideline. The analytical response showed good linearity over the evaluated concentration range (R^2^ = 0.9992), acceptable repeatability (RSD 0.52–4.06%), and consistent recovery (81.84–102.56%) across all formulation matrices. Measurement uncertainty was evaluated as an expanded uncertainty of approximately 10%, indicating controlled variability of quantitative results. Although formulation-dependent differences in analytical response were observed, these effects did not substantially affect quantitative performance. It should be noted that only one commercial product was evaluated per dosage form in this study; thus, the primary focus was on formulation-dependent analytical behavior rather than on capturing product-to-product variability within each dosage form. Overall, the optimized HPLC–PDA analytical strategy provides reliable 5-MTHF quantification across diverse formulation matrices (tablets, chewable tablets, powders, and liquids) using a single harmonized workflow without matrix-specific adjustments. It features simplified room-temperature sample preparation that avoids thermal degradation risks while delivering acceptable recovery (82–103%) and precision (RSD < 4.1%). Measurement uncertainty of ~10% further confirms its suitability for regulatory quality control and routine monitoring of 5-MTHF in fortified foods and health functional foods.

## Figures and Tables

**Table 1:** Comparison of chromatographic conditions of three high-performance liquid chromatography with photodiode array detection methods for the determination of (6S)-5-methyltetrahydrofolate (5-MTHF) using standard solutions.

Method	Mobile Phase Composition	Buffer/ pH Control	R^2^
MFDS [15]	(A) 0.05 M KH_2_PO_4_ (pH 6.5) (B) 0.03 M KH_2_PO_4_ in 35% ACN (pH 8.0)	Required	0.9984
Liu et al. [16]	(A) 0.1% TFA in water (B) Methanol	Not required	0.9997
Alshishani et al. [17]	(A) 50 mM ammonium acetate (B) Methanol	Required	0.9990

**Table 2:** Recovery and precision of (6S)-5-methyltetrahydrofolate (5-MTHF) obtained using different sample preparation procedures across formulation matrices (*n* = 3).

Matrix	Procedure	Concentration (μg/mL)	Mean ± SD (μg/mL)	RSD (%)	Recovery (%)
Tablet	MFDS [15]	12.5	10.53 ± 0.33	3.10	84.28
		25	24.41 ± 0.91	3.71	97.66
		50	41.25 ± 0.42	1.02	82.49
	Liu et al. [16]	12.5	11.92 ± 0.04	0.35	95.38
		25	23.89 ± 0.18	0.79	95.56
		50	48.28 ± 0.37	0.78	96.56
	Alshishani et al. [17]	12.5	12.60 ± 0.39	3.10	100.78
		25	24.57 ± 0.78	3.16	98.30
		50	47.24 ± 1.14	2.41	94.49
Chewable tablet	MFDS [15]	12.5	12.57 ± 0.37	2.98	100.60
		25	24.97 ± 0.22	0.89	99.87
		50	46.51 ± 0.52	1.12	93.01
	Liu et al. [16]	12.5	12.29 ± 0.56	4.55	98.32
		25	25.76 ± 0.95	4.00	95.03
		50	48.96 ± 1.47	2.99	97.91
	Alshishani et al. [17]	12.5	13.10 ± 0.11	0.86	104.76
		25	26.95 ± 0.32	1.18	107.80
		50	51.50 ± 0.98	1.90	102.99
Powder	MFDS [15]	12.5	11.35 ± 0.42	3.73	90.83
		25	25.00 ± 0.57	2.29	99.99
		50	44.70 ± 1.17	3.83	89.40
	Liu et al. [16]	12.5	12.15 ± 0.45	3.68	97.21
		25	24.89 ± 0.79	3.16	99.86
		50	50.61 ± 1.12	2.22	101.23
	Alshishani et al. [17]	12.5	12.21 ± 0.24	1.96	97.67
		25	23.99 ± 0.58	2.41	95.98
		50	49.70 ± 1.34	2.70	99.41
Liquid	MFDS [15]	12.5	10.89 ± 0.48	4.38	87.10
		25	22.83 ± 0.77	3.38	91.34
		50	46.73 ± 0.68	1.46	93.46
	Liu et al. [16]	12.5	12.38 ± 0.24	1.91	99.06
		25	25.27 ± 0.40	1.60	101.09
		50	49.31 ± 0.90	1.83	98.62
	Alshishani et al. [17]	12.5	12.64 ± 0.28	2.24	101.15
		25	25.22 ± 0.87	3.45	100.87
		50	52.44 ± 1.25	2.38	104.88

SD, standard deviation; RSD, relative standard deviation.

**Table 3:** Validation parameters of the optimized HPLC–PDA method for the determination of (6S)-5-methyltetrahydrofolate (5-MTHF).

Analyte	Range (μg/mL)	Slope	Intercept	R^2^	LOD (μg/mL)	LOQ (μg/mL)
5-MTHF	6.25–100	94,651.14	−56,323.78	0.9992	0.15	0.45

**Table 4:** Individual uncertainty components and expanded uncertainty for the determination of 5-methyltetrahydrofolate (5-MTHF) by HPLC–PDA according to the Eurachem Guide.

Analyte	*U*_prep_	*U*_RM_	*U*_std_	*U*_cal_	*U*_rep_	*U*
5-MTHF	0.00271	0.0204	0.00237	0.0397	0.0204	0.0985

## Data Availability

The original contributions presented in this study are included in the article. Further inquiries can be directed to the corresponding authors.

## Abbreviations

The following abbreviations are used in this manuscript:

5-MTHF	(6S)-5-methyltetrahydrofolate
HPLC–PDA	High-performance liquid chromatography–photodiode array
MFDS	Ministry of Food and Drug Safety
ICH	International Council for Harmonisation
AOAC	AOAC INTERNATIONAL
LOD	Limit of detection
LOQ	Limit of quantification
RSD	Relative standard deviation
SD	Standard deviation
TFA	Trifluoroacetic acid
ACN	Acetonitrile
KH_2_PO	Potassium dihydrogen phosphate
